# *Haemophilus parasuis* Subunit Vaccines Based on Native Proteins with Affinity to Porcine Transferrin Prevent the Expression of Proinflammatory Chemokines and Cytokines in Pigs

**DOI:** 10.1155/2013/132432

**Published:** 2013-11-21

**Authors:** R. Frandoloso, S. Martínez-Martínez, E. F. Rodríguez-Ferri, S. Yubero, D. Rodríguez-Lázaro, M. Hernández, C. B. Gutiérrez-Martín

**Affiliations:** ^1^Unidad de Microbiología e Inmunología, Departamento de Sanidad Animal, Facultad de Veterinaria, Universidad de León, 24007 León, Spain; ^2^Laboratório de Imunologia e Doenças Infecto-Contagiosas, Faculdade de Agronomia e Medicina Veterinária, Universidade de Passo Fundo, 99052-900 Passo Fundo, RS, Brazil; ^3^Instituto Tecnológico Agrario de Castilla y León (ITACyL), 47071 Valladolid, Spain

## Abstract

The expression of chemokines (CCL-2 and CXCL-8) and cytokines (IL-1**α**, IL-1**β**, IL-6, TNF-**α**, and IL-10) was evaluated by RT-qPCR in colostrum-deprived pigs vaccinated and challenged with *Haemophilus parasuis* serovar 5. Two vaccines containing native proteins with affinity to porcine transferrin (NPAPTim and NPAPTit) were tested, along with two control groups: one inoculated with PBS instead of antigen (challenge group (CHG)), and another one nonimmunized and noninfected (blank group). The use of NPAPTim and NPAPTit resulted in complete protection against *H. parasuis* (no clinical signs and/or lesions), and both vaccines were capable of avoiding the expression of the proinflammatory molecules to levels similar to physiological values in blank group. However, overexpression of all proinflammatory molecules was observed in CHG group, mainly in the target infection tissues (brain, lungs, and spleen). High expression of CCL-2, CXCL-8, IL-1**α**, IL-1**β**, and IL-6 can be considered one of the characteristics of *H. parasuis* infection by serovar 5.

## 1. Introduction

During infection *Haemophilus parasuis *needs to establish replicative niches in a host that possesses a robust innate immune system, which is an evolutionary ancient form of host defense in multicellular organisms [1]. When *H. parasuis* succeeds in overcoming this response via depletion of specific cell populations [2], Glässer's disease, a severe systemic disorder, develops in pigs.

Proinflammatory cytokines, such as interferon-*α* (IFN-*α*), tumour necrosis factor-*α* (TNF-*α*), interleukin-1 (IL-1), IL-6, and IL-8, are produced during the acute stage of bacterial infection [3–5] and play a pivotal role in the modulation and orchestration of the innate immune response. Each of these molecules has a number of different local and systemic effects, such as macrophage activation [6], increase in adhesion molecules on the vascular endothelium [7, 8], induction of acute phase proteins [9], and neutrophil chemotaxis and activation [10]. Some bacterial molecules as LPS can induce the production of proinflammatory cytokines, which results in fever, hypotension, inadequate tissue perfusion, metabolic acidosis, septic shock, and organ failure [11]. In order to regulate the severity of the inflammatory response, immune system has some cytokines, such as IL-10, which are capable of inhibiting a large amount of cytokines and chemokines secreted by macrophages and activated monocytes [12, 13].

Little is known about the interaction between *H. parasuis* and cytokine expression in pigs, which hinders the understanding of Glässer's disease pathogenesis and the development of effective vaccines. We evaluated in this study the capability of a subunit vaccine to inhibit the transcription of a set of chemokines and cytokines directly related to inflammation, in systemic and target infection organs. Colostrum-deprived pigs [14] were used as animal model in order to avoid the endemic colonization by *H. parasuis* in the upper respiratory tract of suckling piglets that have contact with their sows.

## 2. Materials and Methods

### 2.1. Vaccines, Experimental Groups and Immunization

Two vaccines based on native proteins with affinity to porcine transferrin (NPAPTim and NPAPTit) [15] were compared. NPAPTim contained 400 *μ*g of NPAPT antigen adjuvanted with Montanide IMS 2215 VG PR (Seppic Inc., Paris, France) in a 1 : 4 ratio. NPAPTit contained the same antigen but potentiated with neuraminidase from *Clostridium perfringens *(type VI) at a concentration of 100 mU/mL. Twenty colostrum-deprived pigs were randomly assigned to four experimental groups. The NPAPTim group (*n* = 6) received 2 mL of NPAPTim vaccine by intramuscular injection at 28 and 49 days of age, while the NPAPTit group (*n* = 6) received the same volume of NPAPTit vaccine by intratracheal injection at the same days. The challenge control (CHG) group (*n* = 4) received 2 mL of PBS intramuscularly at the same days. These three groups were challenged intratracheally at 63 days with a lethal dose (3 × 10^8^ CFU) of *H. parasuis *Nagasaki strain (serovar 5). The fourth group (*n* = 4) comprised pigs that were maintained as nonimmunized, noninfected blank group. All experiments were conducted in accordance to the guidelines of the University of León Ethical Committee. Surviving pigs were humanely euthanized at 78 days. More details of this schedule have been already reported [15].

### 2.2. Sample Collection and RNA Extraction

This description is based on the MIQE guidelines [16]. After necropsy, small pieces (≤0.5 cm) from brain, lungs, spleen, and mediastinal, tracheobronchial, and mandibular nodes from each pig were collected aseptically and maintained in RNA later solution (Ambion-Applied Biosystems, Life Technologies, USA) overnight at 4°C. After centrifugation, the supernatant was discarded, and the samples were stored at −80°C. Total cellular RNA was purified from 30 mg of tissue of each sample, using the RNAspin Mini RNA Isolation Kit (GE Healthcare, Spain). Samples were maintained at −80°C in 40 *μ*L of RNase free water. Immediately before performing the cDNA synthesis, the concentration and quality (integrity) of the total RNA were checked by a capillary electrophoresis system, using the Agilent 2100 Bioanalyzer and the RNA 6000 Pico Chip Kit. All RNA obtained had a RNA integrity number (RIN) higher than 7 (scale 1 to 10).

### 2.3. cDNA Synthesis

The first-strand cDNA synthesis was carried out by reverse transcription (RT) from total RNA using the SuperScript III First-Strand Synthesis SuperMix (Invitrogen Life Technologies, USA). Briefly, 2 *μ*g of total RNA was primed with 1 *μ*L of random hexamers (50 ng/*μ*L) and 1 *μ*L of annealing buffer and was completed until 8 *μ*L with RNase free water. It was incubated at 65°C for 5 min and then immediately placed on ice for 1 min. Afterwards, 10 *μ*L of 2 × First-Strand Reaction Mix and 2 *μ*L of SuperScript III/RNaseOUT Enzyme Mix were added to the tube on ice and incubated at 25°C for 10 min, at 50°C for 50 min and at 85°C for 5 min, to stop the reaction. The resulting cDNA was diluted 10 times and stored at −80°C until use.

### 2.4. Primers and Probes Design

The set of primers and hydrolysis probes used to determine the expression of chemokines (CCL2 and CXCL-8), cytokines (IL-1*α*, IL-1*β*, IL-6, TNF-*α*, and IL-10), and reference genes (GAPDH, *β*-actin, cyclophilin) were designed for this study or obtained from previous reports (Table 1). Hydrolysis probes were labeled with 6-carboxy-fluorescein (FAM) at the 5′ end and the Black Hole Quencher (BHQ_1_ or BHQ_2_) at the 3′ end.

### 2.5. Development of a PCR Internal Control

The cDNA obtained from tracheobronchial nodes of CHG group was amplified using the primers described in Table 1, including a final step of 72°C for 30 min. The PCR-specific product from every gene was cloned into the pGEM-T-Easy vector (Promega, Spain) according to the manufacturer's instructions.

### 2.6. Quantitative Real-Time PCR (qPCR) Analysis

Quantitative real-time PCRs were carried out in 96-well plates (LightCycler 480 Multiwell Plates 96, white, -Roche-) in a total volume of 20 *μ*L containing 500 nM of each primer, 100 nM of each specific probe, 1 × Light Cycler Probes Master (Roche) and 5 *μ*L of cDNA. The plates were sealing with an adhesive foil (Roche), and then the PCRs were run on a LightCycler 480 II System (Roche) using the following thermocycling conditions: an initial denaturation step (95°C, 10 min) followed by 50 cycles of 95°C for 10 s (denaturation), 60°C for 30 s (annealing), and 72°C for 30 s (extension). qPCRs were run in triplicate for better accuracy and reproducibility. No template controls (NTC) from RT and qPCR were additionally included. Calibration curves for each chemokine, cytokine and reference gene were obtained using tenfold serial dilutions of a plasmid DNA containing the specific nucleotide sequence (PCR internal control) of each gene. The relative quantification of each gene was calculated automatically by LightCycler Relative Quantification Software, using the ΔΔCT-Method (Roche). The PCR efficiencies are shown in Table 1.

### 2.7. Statistical Analysis

NPAPTim, NPAPTit, and blank groups, whose pigs remained alive until the end of the experiment, were compared with each other. CHG group was not included in the comparative analysis because these animals died at a different time point. Descriptive statistics (mean, standard error), normality (Shapiro-Wilk), and homoscedasticity (Bartlett's test) were determined. The analysis of cytokines expression levels was performed using one-way analysis of variance (ANOVA). Statistical means were contrasted by Tukey test (*P* < 0.05) using the Statistix 8.0 (Analytical Software, Roseville, MN, USA) [17]. The GraphPad Prism statistical program, version 5.0 (San Diego, CA, USA) was used for the figures.

## 3. Results and Discussion

### 3.1. Clinical Results

All pigs from the NPAPTim and NPAPTit groups survived until the end of experiment, and no clinical symptoms were observed. As could be expected, all pigs from CHG group died after the challenge, showing the typical clinical symptoms of Glässer's disease: high temperature, limb incoordination, swollen joints, severe dyspnea, and coughing. Clinical signs and histopathological findings have been extensively reported in Frandoloso et al. [15].

### 3.2. Evaluation of Endogenous Gene Expression

Three reference genes were tested due to different cell composition of the organs compared (brain, lungs, spleen, and lymph nodes). Cyclophilin showed constantly the highest homogeneous level of expression in all organs, with an average quantification cycle (Cq) of 21.5 ± 1.5, whereas *β*-actin and GAPDH expression levels were lower and less constant in all tissues, with a Cq mean of 26.0 ± 2.2 and 27.0 ± 3.3, respectively. On the basis of these results, cyclophilin was used as reference gene for normalization of data.

### 3.3. Chemokine Expression

**Figure 1:**
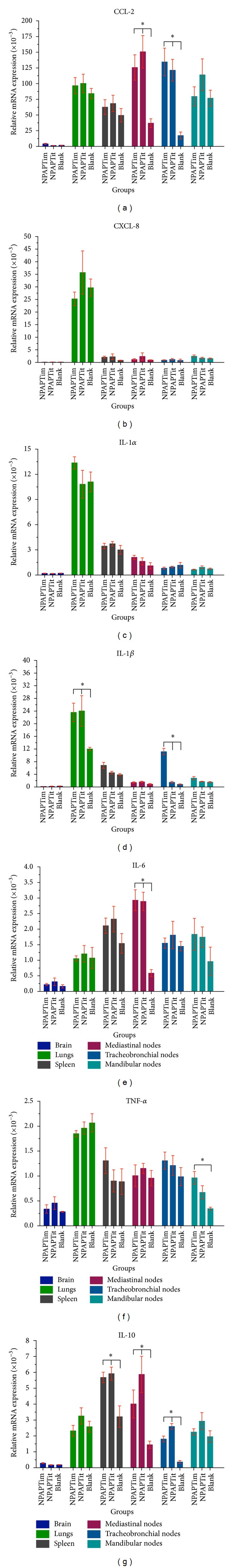
Relative mRNA expression rates of chemokines and cytokines from different tissues in NPAPTim, NPAPTit and blank groups. *indicates significant differences between groups (*P* < 0.05).

No significant changes in transcript levels of CCL-2 (Figure 1(a)) and CXCL-8 (Figure 1(b)) genes were detected between NPAPTim, NPAPTit, and blank groups. There were only two exceptions: that of mediastinal and that of tracheobronchial nodes, for which the NPAPT groups showed a CCL-2 expression of about three- and seven-fold higher, respectively, than that of blank group (*P* < 0.05). However, CHG group transcribed high CCL-2 amounts in all tissues (Table 2). In lungs and draining lymph nodes, the CCL-2 transcription was approximately 20 and 50 times higher, respectively, than that of blank group. The origin of this chemokine could be justified by the activation of alveolar macrophages after *H. parasuis* contact or by the damage induced by this pathogen on epithelial cells. In the brain of CHG pigs, a high CCL-2 level was also observed (approximately 150 times higher than those of NPAPT and blank groups, Figure 1(a) and Table 2), which could be related to the presence of macrophages among the inflammatory cells seen in the histopathological study of this organ, whose lesions were compatible with meningitis [15]. Similarly, increased CCL-2 levels in the spleen and lymph nodes in CHG group seem to suggest a cellular proinflammatory immune response related to the activation of resident macrophages and dendritic cells, because the recruitment of inflammatory monocytes is dependent mainly on CCL-2 [18]. This overall increase in CCL-2 levels was coincident and could explain the elevation in the number of activated monocytes (CD172*α* ^+^CD163^+^) in the peripheral blood [2].

CXCL-8 has been characterized as the main neutrophil activating chemoattractant chemokine [10]. The high CXCL-8 level measured in the brain of CHG pigs (about 50 times higher than that of blank group, Figure 1(b) and Table 2) could explain the great amount of neutrophils present in inflammatory infiltrates of this organ, as it has been already reported [19]. Bouchet et al. [19, 20] tested the *in vitro* ability of *H. parasuis* to induce IL-8 expression in porcine tracheal and brain vascular microendothelial cells and showed that both dead and alive *H. parasuis* were capable of producing high IL-8 levels. In our *in vivo* study, a correlation could be established between the severity of the suppurative meningitis [15] and the brain CXCL-8 expression. We also observed that the CXCL-8 expression after *in vivo* infection was very similar to that reported for the *in vitro* experiment by Bouchet et al. [19, 20]. This result could indicate that the number of *H. parasuis *organisms reaching the brain was also similar to that used in *in vitro* infection, thus suggesting for the first time that *H. parasuis* could multiply and disseminate very fast in the natural host and that the *in vitro* model using brain and tracheal cells is adequate to perform studies about the expression of proinflammatory molecules. Identical with CCL-2 expression, CXCL-8 was also more transcribed in the tissues associated with the site of inoculation of *H. parasuis* (Table 2).

### 3.4. Cytokine Expression

IL-1*α* levels were quite similar for all groups, except for CHG animals (Figure 1(c) and Table 2). However, the two vaccinated groups expressed significantly higher IL-1*β* levels than that of the blank group in lungs (*P* < 0.05, Figure 1(d)). A significantly higher rise in IL-1*β* expression was also observed in tracheobronchial nodes in NPAPTim group (*P* < 0.05). A previous study reported by our group [5] showed a high IL-1*α* expression in the brain of a pig dying as a consequence of Glässer's disease. In the current report, we wanted to know how IL-1*β* responded to infection, and our results allowed us to assure that pigs suffering from Glässer's disease expressed high amounts of this cytokine. The greatest IL-1*β* expression in CHG group was measured in lungs, where the levels were about 50 and 25 times higher respectively than those of blank and NPAPT groups. A more marked difference was observed when comparing the IL-1*β* expression in the brain in CHG group (more than 500 times higher) with that measured in NPAPT groups (Figure 1(d) and Table 2). IL-1*β* expression has been correlated with pathologies in which inflammatory manifestations are seen [21]. Our results suggest that IL-1*α* and IL-1*β* could play an important role in the inflammatory response developed against *H. parasuis*, because both molecules can bind with the same signaling receptor and then induce the propagation of the inflammatory response [21].

The vaccinated groups showed an IL-6 concentration about five times higher in mediastinal nodes than that of blank group (*P* < 0.05, Figure 1(e)). The highest IL-6 expression in these three groups was detected in the secondary lymph tissues, being lower in lungs and especially in brain (Figure 1(e)). CHG group showed considerable IL-6 increases compared to the three other groups, between approximately six and 40 times higher depending on the tissue tested (Figure 1(e) and Table 2). IL-6 has been directly related to the synthesis of acute phase proteins and, in this respect, high levels of pig MAP, C-reactive protein and haptoglobin in the serum have been reported in pigs that died after experimental *H. parasuis* infection [22, 23]. On the other hand, IL-6 plays an important role in Th2 lineage differentiation, being abundantly produced by antigen presenting cells [24]. The increase detected in mediastinal nodes in NPAPT groups could be associated to the stimulation and development of a Th2 response but not to a proinflammatory situation, mainly because low transcriptions of CXCL-8, IL-1 isoforms and TNF-*α* were observed in this tissue. Otherwise, the systemic transcription of high IL-6 levels measured in CHG group during *H. parasuis* pathogenesis could be due to the direct stimulation of this pathogen on mononuclear phagocytes, epithelial cells, and vascular endothelial cells [25].

Significantly lower values (*P* < 0.05) were detected in TNF-*α* expression in blank group in mandibular nodes when compared with any of the vaccinated groups (Figure 1(f)), while in the remaining tissues these levels remained similar. Moreover, the TNF-*α* levels increased markedly in CHG group (Table 2), especially in the secondary lymph tissues, where the expression was more than twelve times higher compared with any of the healthy groups (Figure 1(f) and Table 2). The TNF-*α* values observed in vaccinated and blank pigs suggest the existence of a basal expression level of this cytokine in swine, such as that observed in mice [26] and guinea pigs [27]. Biologically, the combination of TNF-*α* and IL-1 can induce changes in endothelial cells of small vessels, thus increasing the adherence of inflammatory cells. In addition, a TNF-*α* increase could cause edema and vascular thrombosis [28]. These findings allow us to relate the high expression of IL-1 isoforms, and TNF-*α* with the edema, lung congestion and vascular thrombosis observed microscopically in a previous report in CHG pigs [15].

The two vaccinated groups showed transcription levels statistically higher than the blank group for IL-10 in spleen and mediastinal and tracheobronchial nodes (*P* < 0.05, Figure 1(g)). However, these values were at least thrice lower than those measured for CHG group, except for mediastinal nodes (Table 2). Taking into account the regulative effect exerted by IL-10 on the different cells belonging to innate and adaptive systems, it could be suggested that the elevation observed in CHG group would be a regulative action of the secretion of proinflammatory cytokines. On the other hand, Go et al. [29] showed that B cells cultivated *in vitro* and stimulated with IL-10 increased the class II MHC expression on the surface of B cells. The increase of IL-10 expression in spleen in NPAPT pigs could be explained by this fact, mainly because these animals showed hyperplasia in the white pulp, characterized by an increase of the spleen germinal centers and by a peripheral increase in B cells, and in the expression of SLAIIDR molecules [2].

## 4. Conclusion

The experimental infection of colostrum-deprived pigs with *H. parasuis* Nagasaki strain induced an overregulation of the expression of proinflammatory chemokines and cytokines related to innate immunity. Because of the differences in the expression between the protected (NPAPTim and NPAPTit) and nonprotected (CHG) groups, CCL-2, CXCL-8, IL-1*α*, IL-1*β*, and IL-6 may be considered the best inflammatory markers to *H. parasuis* infection in target organs. Finally, the protection conferred by NPAPT vaccines was enough to prevent an inflammatory reaction mediated by CCL-2, CXCL-8, IL-1*α*, IL-1*β*, IL-6, TNF-*α*, and IL-10, making NPAPT antigen a suitable candidate to control Glässer's disease caused by *H. parasuis* Nagasaki strain.

## Figures and Tables

**Table 1:** Oligonucleotides used in qPCR analysis.

Genes	Sense	Sequences (5′ to 3′)	Product	Efficiency (%)	Reference

IL1-*α*	→	GTGCTCAAAACGAAGACGAACC	110 bp	99.5 ± 0.5	Duvigneau et al. [30]
	←	CATATTGCCATGCTTTTCCCAGAA			
	→	**FAM**-TGCTGAAGGAGCTGCCTGAGACACCC-**BHQ1**			

IL1-*β*	→	CCAAAGGCCGCCAAGATATAA	75 bp	100 ± 0.2	Arce et al. [31]
	←	GGACCTCTGGGTATGGCTTTC			
	→	**FAM**-CTGACTTCACCATGGAAGTCCTCTCTCCCTAAG-**BHQ2**			

TLR-4	→	GCCATCGCTGCTAACATCATC	108 bp	99.7 ± 0.2	This study
	←	CTCATACTCAAAGATACACCATCGG			
	→	**FAM**-CAAAAGTCGGAAGGTTATTGTCGTGGTGTC-**BHQ1**			

IL-6	→	CTGGCAGAAAACAACCTGAACC	94 bp	98.6 ± 0.2	Duvigneau et al. [30]
	←	TGATTCTCATCAAGCAGGTCTCC			
	→	**FAM**-TTGAACCCAGATTGGAAGCATCCGTCTTTT-**BHQ1**			

IL-10	→	CGGCGCTGTCATCAATTTCTG	89 bp	100.4 ± 0.5	Duvigneau et al. [30]
	←	CCCCTCTCTTGGAGCTTGCTA			
	→	**FAM**-AGGCACTCTTCACCTCCTCCACGGC-**BHQ1**			

TNF-*α*	→	GCCCTGGTACGAACCCATCTA	91 bp	99.1 ± 0.3	Arce et al. [31]
	←	CAGATAGTCGGGCAGGTTGATCTC			
	→	**FAM**-CCAGCTGGAGAAGGATGATCGACTCAGT-**BHQ2**			

CCL-2	→	ACCAGCAGCAAGTGTCCTAAAG	92 bp	99.4 ± 0.7	Arce et al. [31]
	←	TCCTGGACCCACTTCTGCTT			
	→	**FAM**-AGCAGTGATCTTCAAGACCATCGCGG-**BHQ2**			

CXCL-8	→	TTCGATGCCAGTGCATAAATA	120 bp	99.8 ± 0.8	Arce et al. [31]
	←	TGACAAGCTTAACAATGATTTCTGAA			
	→	**FAM**-CATTCCACACCTTTCCACCCCAAATTTATC-**BHQ2**			

GAPDH	→	ACATGGCCTCCAAGGAGTAAGA	106 bp	99.2 ± 0.6	Duvigneau et al. [30]
	←	GATCGAGTTGGGGCTGTGACT			
	→	**FAM**-CCACCAACCCCAGCAAGAGCACGC-**BHQ1**			

cyclophilin	→	TGCTTTCACAGAATAATTCCAGGATTTA	77 bp	100.0 ± 0.1	Duvigneau et al. [30]
	←	GACTTGCCACCAGTGCCATTA			
	→	**FAM**-TGCCAGGGTGGTGACTTCACACGCC-**BHQ1**			

*β*-actin	→	CTCGATCATGAAGTGCGACGT	114 bp	101.0 ± 0.3	Duvigneau et al. [30]
	←	GTGATCTCCTTCTGCATCCTGTC			
	→	**FAM**-ATCAGGAAGGACCTCTACGCCAACACGG-**BHQ1**			

**Table 2:** Expression of proinflammatory molecules from different tissues in CHG group (mean ± standard error).

Molecules*	Tissue					
	Brain	Lung	Spleen	Mediastinal nodes	Tracheobronchial nodes	Mandibular nodes
CCL-2	204.2 ± 40.2	1673.2 ± 96.5	441.7 ± 118.2	901.5 ± 58.9	948.8 ± 73.8	257.1 ± 45.2
CXCL-8	55 ± 3.6	887.6 ± 34.2	12.7 ± 1.6	431.5 ± 74.3	470.7 ± 104.4	17.3 ± 2.4
IL-1*α*	57.7 ± 5.8	365.4 ± 45.6	35.3 ± 1.7	142.1 ± 20.7	15.3 ± 3.2	15.5 ± 2.1
IL-1*β*	141.5 ± 11.4	615.4 ± 76.1	92.7 ± 2.8	184.1 ± 32.2	119.2 ± 13.1	63 ± 12.2
IL-6	2.5 ± 0.4	37.4 ± 5.9	20.8 ± 3.7	35.8 ± 1.8	115.1 ± 20.42	13.6 ± 0.7
TNF-*α*	10.5 ± 0.7	10.9 ± 1.1	19 ± 3.2	75.1 ± 5.4	22.2 ± 4.3	16.5 ± 0.6
IL-10	17.5 ± 1.7	48.1 ± 6.6	10.8 ± 1.8	6.4 ± 1.7	7.1 ± 0.8	12.1 ± 1.5

*Relative mRNA expression (×10^−3^).
